# Age-Dependency of Neurite Outgrowth in Postnatal Mouse Cochlear Spiral Ganglion Explants

**DOI:** 10.3390/brainsci10090580

**Published:** 2020-08-21

**Authors:** Claudia Frick, Stefan Fink, Dominik Schmidbauer, Francis Rousset, Holger Eickhoff, Anke Tropitzsch, Benedikt Kramer, Pascal Senn, Rudolf Glueckert, Helge Rask-Andersen, Karl-Heinz Wiesmüller, Hubert Löwenheim, Marcus Müller

**Affiliations:** 1 Department of Otolaryngology, Head and Neck Surgery, Tübingen Hearing Research Centre, University of Tübingen Medical Center, 72076 Tübingen, Germany; claudia.frick@tuebingen.mpg.de (C.F.); anke.tropitzsch@med.uni-tuebingen.de (A.T.); benedikt.kramer@umm.de (B.K.); hubert.loewenheim@med.uni-tuebingen.de (H.L.); marcus.mueller@uni-tuebingen.de (M.M.); 2 Department of Microbiome Science, Max Planck Institute for Developmental Biology, 72076 Tübingen, Germany; 3 Inner Ear Laboratory Innsbruck, Medical University Innsbruck, 6020 Innsbruck, Austria; dominik.schmidbauer@i-med.ac.at (D.S.); rudolf.glueckert@i-med.ac.at (R.G.); 4 The Inner Ear & Olfaction Lab, Department of Clinical Neurosciences, Faculty of Medicine, University of Geneva, 1206 Geneva, Switzerland; francis.rousset@unige.ch (F.R.); pascal.senn@hcuge.ch (P.S.); 5 EMC Microcollections GmbH, 72070 Tübingen, Germany; eickhoff@microcollections.de (H.E.); wiesmueller@microcollections.de (K.-H.W.); 6 Department of Otolaryngology, Head and Neck Surgery, University Hospital Mannheim, 68167 Mannheim, Germany; 7 Tirol Kliniken Innsbruck, University Clinic of Otolaryngology, 6020 Innsbruck, Austria; 8 Department of Surgical Sciences, Otorhinolaryngology and Head and Neck Surgery, University of Uppsala, 751 85 Uppsala, Sweden; helge.raskandersen@gmail.com

**Keywords:** small-molecule BDNF mimetics, THF, NT-3, Trk receptors, SGN, murine postnatal model, spiral ganglion explant culture, neurite outgrowth, in vitro, NMRI mice

## Abstract

Background: The spatial gap between cochlear implants (CIs) and the auditory nerve limits frequency selectivity as large populations of spiral ganglion neurons (SGNs) are electrically stimulated synchronously. To improve CI performance, a possible strategy is to promote neurite outgrowth toward the CI, thereby allowing a discrete stimulation of small SGN subpopulations. Brain-derived neurotrophic factor (BDNF) is effective to stimulate neurite outgrowth from SGNs. Method: TrkB (tropomyosin receptor kinase B) agonists, BDNF, and five known small-molecule BDNF mimetics were tested for their efficacy in stimulating neurite outgrowth in postnatal SGN explants. To modulate Trk receptor-mediated effects, TrkB and TrkC ligands were scavenged by an excess of recombinant receptor proteins. The pan-Trk inhibitor K252a was used to block Trk receptor actions. Results: THF (7,8,3′-trihydroxyflavone) partly reproduced the BDNF effect in postnatal day 7 (P7) mouse cochlear spiral ganglion explants (SGEs), but failed to show effectiveness in P4 SGEs. During the same postnatal period, spontaneous and BDNF-stimulated neurite outgrowth increased. The increased neurite outgrowth in P7 SGEs was not caused by the TrkB/TrkC ligands, BDNF and neurotrophin-3 (NT-3). Conclusions: The age-dependency of induction of neurite outgrowth in SGEs was very likely dependent on presently unidentified factors and/or molecular mechanisms which may also be decisive for the age-dependent efficacy of the small-molecule TrkB receptor agonist THF.

## 1. Introduction

Cochlear implants (CIs), which are surgically placed into the cochlea, bypass damaged structures in the inner ear by directly stimulating the auditory nerve. Due to a spatial gap between spiral ganglion neurons (SGNs) of the auditory nerve and the CI electrodes, which mostly lie along the outer wall of the scala tympani [1,2,3], CI performance is limited by a broad spread of electrical charge [4] that limits frequency selectivity. Eliminating this spatial gap by promoting SGN survival and fiber growth toward and onto the CI electrode array is a generally accepted strategy for the improvement of CI performance (for review, see [5]).

For SGN survival and fiber growth during cochlear development, two members of the neurotrophin family, namely, brain-derived neurotrophic factor (BDNF) and neurotrophin-3 (NT-3), and their tropomyosin receptor kinases B (TrkB) and C (TrkC) are required [6,7,8,9,10]. BDNF and NT-3 significantly stimulate SGN survival and fiber growth when applied to cultured SGNs in vitro [11,12,13,14,15] or administered to the deafened cochlea in vivo (for review, see [16]). However, clinical application of neurotrophins is limited by poor pharmacokinetic properties because of high biodegradability and short plasma half-life [17].

To circumvent this problem, compounds that mimic the neurotrophic activity of BDNF, but possess superior pharmacokinetic properties, were identified. Based on in silico [18] and cell-based screenings [19,20,21], several promising small-molecule TrkB receptor agonists, including LM22A-1, LM22A-4, deoxygedunin (DG), 7,8-dihydroxyflavone (DHF), and 7,8,3′-trihydroxyflavone (THF), were described (for review, see [22]). Two of these novel small molecules, namely, DHF and THF, promoted SGN survival in vitro and in vivo and stimulated neurite outgrowth in organotypic cochlear cultures of postnatal day 1 (P1) and P2 mouse cochleae [23,24]. A detailed structural-activity relationship study on the flavonoids revealed the catechol group (7,8-dihydroxy on the A ring) of DHF and THF to be indispensable for the agonistic activity on the TrkB receptor [21]. As responsiveness to neurotrophins differs with age [11,15,25,26], differential responsiveness to small-molecule TrkB agonists also appears to be very likely, which requires attention when developing therapeutic strategies utilizing “immature” model systems. All cell culture systems, as well as organotypic cultures, do not exactly reflect the adult situation, but are crucial for drug screening purposes. For targeted pharmacological manipulations, the understanding of developmental processes is an indispensable prerequisite to gain insights into molecular mechanisms which are necessary for the stimulation of neurite outgrowth in SGNs.

To investigate neurite outgrowth in SGNs, we used spiral ganglion explants (SGEs), which serve as a well-accepted and commonly used model [25,27,28,29,30,31,32] that allows to study SGNs in their natural tissue formation in a controllable setting. As BDNF and NT-3 are differentially expressed in SGNs themselves [33,34], we investigated differences in spontaneous neurite outgrowth in SGEs from P3, P4, P5, P6, and P7 mouse cochlea. Likewise, age-dependent differences in responsiveness to BDNF were evaluated using SGEs from P3 to P7 mouse cochlea. To analyze age-dependent differences in small-molecule TrkB agonist efficacy, neurite outgrowth in P4 and P7 SGEs was compared. As BDNF and NT-3 are differentially expressed in peripheral targets of SGNs [35,36], we tested whether those neurotrophins and compounds released from the organ of Corti affect neurite outgrowth and small-molecule TrkB agonist efficacy. To gain insights into the underlying molecular mechanisms, Trk receptor function was blocked by the pan-Trk inhibitor K252a [37] and Trk receptor ligands were scavenged by recombinant TrkB/TrkC proteins [38].

## 2. Materials and Methods

### 2.1. Animals

SGEs were dissected from cochleae of 285 postnatal NMRI mice of both genders. Mice were obtained from Charles River (Sulzfeld, Germany) and bred in an in-house animal facility. All animals received care in accordance with the standards described by the German ‘Law on Protecting Animals’ (Tierschutzgesetz) and with the European Directive 2010/63/EU for protection of animals used for experimental purposes. Harvesting of the cochleae was approved by local authorities (Regierungspräsidium Tübingen, applications dated 16, August, 2012, 19, December, 2013, and 26, March, 2018) in accordance with the guidelines regarding the care and use of experimental animals.

### 2.2. Harvesting of Spiral Ganglion Explants

P3 to P7 NMRI mice were used. Pups were killed by decapitation with sterile scissors. The heads were placed in a cell culture dish with 70% ethanol and transferred into a laminar flow cabinet (Heraeus^®^ HERAsafe^®^, Heraeus Holding GmbH, Hanau, Germany). All further steps were performed under sterile conditions. After dabbing the heads dry, the skin was removed and the skull was sectioned along the mid-sagittal plane. The brain was removed and both half-skulls were immersed in ice-cold CaCl_2_/MgCl_2_-containing Hank’s Balanced Salt Solution (HBSS; Gibco/Life Technologies, Karlsruhe, Germany). The cochleae were removed under a stereo microscope (Leica MZ-6; Leica Mikrosysteme Vertrieb GmbH, Wetzlar, Germany) and transferred into a cell culture dish with ice-cold HBSS. The cartilaginous cochlear capsule was opened and the stria vascularis and the organ of Corti (OC) were removed in one piece. For experiments which included OC explants, the OC was separated from the stria vascularis by pulling these parts carefully apart. To obtain SGEs, the apical turn of the cochlea was cut off. Remaining osseous debris was removed and the most apical part of the cochlea was cut into two pieces of about 500 µm in size. Residual axonal processes were truncated, thereby separating the spiral ganglia from the modiolus.

### 2.3. Treatment and Culture of Spiral Ganglion Explants

SGEs were cultured in 8-well culture slides (Corning^®^ Biocoat™ Poly-D-Lysine/Laminin 8-Well Culture Slides; Corning Incorporated, New York, USA). Culture medium was freshly prepared prior to cochlear dissection and consisted of 9.44 mL Neurobasal^®^ medium, 100 µL HEPES buffer solution (10 mM), 250 µL L-glutamine (5 mM), and 200 µL B-27^®^ supplement 50x (Gibco/Life Technologies). To prevent bacterial contamination, 10 µL penicillin (100 U/mL) was added (Sigma-Aldrich, St. Louis, USA). BDNF and NT-3 (R&D Systems, Minneapolis, USA) were dissolved in medium at concentrations of 25 ng/mL, 3 ng/mL, and 1 ng/mL. The TrkB agonists, LM22A-1, LM22A-4 (both from EMC microcollections GmbH, Tübingen, Germany), deoxygedunin (DG; Gaia Chemical Corporation, New Milford, USA), 7,8-dihydroxyflavone (DHF; TCI Deutschland GmbH, Eschborn, Germany), and 7,8,3′-trihydroxyflavone (THF; abcr GmbH, Karlsruhe, Germany), were dissolved at a concentration of 1 µM. Recombinant proteins TrkB/Fc and TrkC/Fc (R&D Systems) were used at a concentration of 1 µg/mL. K252a was applied at a concentration of 100 nM. Each well was filled with 200 µL of complemented medium and populated with a single SGE or one SGE and two OC explants. To prevent damage of the primary tissues, explants were transferred using a micro spatula with spoon ends. To avoid direct contact between the SGE and OC explants, OC explants were placed a few millimeters apart. To ensure direct contact with the substrate, explants were carefully pushed to the bottom of the culture slide. Culture slides were transferred into an incubator (Heraeus^®^ HERAcell^®^, Heraeus Holding GmbH, Hanau, Germany) and explants were cultured for 96 h (4 days in vitro (DIV)) at 37 °C in 5% CO_2_ atmosphere. Each experiment was performed in triplicate at minimum.

### 2.4. Fixation and Immunostaining

Cultured explants were fixed using Roti^®^-Histofix 4% phosphate-buffered formaldehyde solution (Carl Roth GmbH + Co. KG, Karlsruhe, Germany) for 20 min at room temperature (RT). The fixing solution was removed and explants were washed with phosphate-buffered saline (PBS; PBS tablets, Gibco/Life Technologies) supplemented with 0.2% Triton-X 100 (Merck Chemicals GmbH, Schwalbach, Germany). While explants were incubated in the washing solution, a blocking solution was prepared. Bovine serum albumin (BSA, 1%; Sigma-Aldrich, St. Louis, USA) was dissolved in PBS/0.2% Triton-X 100. The washing solution was removed and the blocking solution was applied to the explants for 60 min at RT. Immunostaining of neurons and their processes was performed using a polyclonal mouse anti-neurofilament 200 antibody (NF200, 1:800; Sigma-Aldrich). The integrity of the OC was analyzed with a polyclonal rabbit anti-myosin VIIA antibody (MyoVIIA, 1:500; Proteus Biosciences Inc., Ramona, CA, USA). Antibodies were diluted in PBS/0.1% Triton-X 100 supplemented with 0.5% BSA. Incubation was carried out overnight at 4°C. The next morning, antibody solution was removed and explants were washed with the washing solution three times. Visualization of the primary antibodies was performed with an Alexa 488-conjugated goat anti-mouse and an Alexa 546-conjugated goat anti-rabbit secondary antibody (1:400 in PBS/0.1% Triton-X 100/0.5% BSA; Molecular Probes/Life Technologies GmbH). The secondary antibody was incubated for 60 min at RT. Antibody solution was removed and explants were washed three times with the washing solution. Nuclei were counterstained using 300 nM DAPI (4′,6-diamidino-2-phenylindole; Life Technologies GmbH) which was incubated for 20 min at RT. DAPI solution was removed and explants were washed once. After removal of the washing solution, culture slide walls were removed using the provided plastic device. Explants were mounted in FluorSave™ Reagent (Merck Chemicals GmbH), covered with a cover slip (R. Langenbrinck, Emmendingen, Germany), and kept in opaque boxes at 4^°C^ for storage.

### 2.5. Imaging

Digital images were obtained using a Zeiss Axio Imager M2 fluorescence microscope equipped with a motorized stage (Carl Zeiss Microscopy GmbH, Oberkochen, Germany). Using the ZEN Tiles & Positions module with a 10x objective, an overview of the entire neurite outgrowth was depicted. An overlap of 10% between single images was used. Images were converted to JPG format for analysis.

### 2.6. Analysis of Neurite Outgrowth

Neurite outgrowth was quantified using ImageJ (Version 1.47v; Wayne Rasband, National Institutes of Health, USA; [39]). First, images were scaled by a factor of 0.65 to equate the number of pixels with the distance (1 pixel = 1 µm). Images were converted to binary 8-bit images. A standardized algorithm was developed for consistent image editing. A median filter “Despeckle” was used to reduce noise. The “Dilate” command added pixels to the edges of black objects which broadened fine neurites and helped to preserve thin neuronal processes. Staining artifacts, debris, and neuronal somata were manually removed using the eraser tool. To quantify neurite outgrowth, a Sholl analysis was utilized [40]. Based on this method, a customized plugin was created (code available on request) that computed a “neurite length index”, which represents the entire neurite outgrowth originating from one SGE [41,42]. In brief, the algorithm required to set a midpoint in the middle of the explant to generate circles with increasing radii (radius step size: 10 µm). The number of intersections of the circles with the neurites were counted pixel-wise and averaged for 250 µm radius bins. Neurite length was calculated by multiplying the mean number of intersections with the distance from the midpoint, beginning at the largest, outermost radius bin. Progressing toward smaller radii, the mean number of intersections already processed was subtracted from the number of intersections of the previous radius bin (to avoid overestimations), before being multiplied with the distance. Single neurites were represented by few to individual pixels along their extension counted in the different radius bins. Based on this pixel-wise counting, multiple neurites running alongside each other and forming bundles did not have to be segregated for analysis. Instead, the increased number of pixels counted in each radius bin for such fascicles contributed appropriately to the increased number of fibers. Overall, the neurite length index (NLI in µm) was calculated by determining the sum of all relative neurite length values obtained. To investigate neurite number and extension, histograms of the number of intersections were plotted as a function of the distance from the midpoint. Mean numbers of intersections at 500 µm and 1500 µm distance were analyzed. SGEs with an NLI of 0 were excluded from data analysis.

### 2.7. Statistical Analysis

The JMP software (JMP^®^ 11.0.0 (64-bit); SAS Institute Inc., Cary, NC, USA) was used for data analysis. Distribution patterns of neurite length indices did show log-normal distribution. The approximation to a normal distribution was achieved by logarithmic transformation of the data. As previously described [43], the statistical analysis was performed on transformed data and the obtained values were transformed back at the end of the analysis for graphical output. The statistical analysis was performed on logarithmized NLI group mean values using one- or two-way ANOVA with Dunnett or Tukey–Kramer post-hoc test, depending on whether means were compared to the same control group or all possible pairs of means were compared. Data were presented as arithmetic mean ± one standard deviation (for the mean number of intersections) or as the geometric mean ± the upper and lower 95% confidence intervals (for the NLI values in µm), stated as, for example, NLI of 2186 (±1722/964; *n* = 15), where *n* represents the number of evaluated explants. The level of significance was set at *p*-value < 0.05.

## 3. Results

### 3.1. Age-Dependent Differences in Spontaneous Neurite Outgrowth

**Figure 1:**
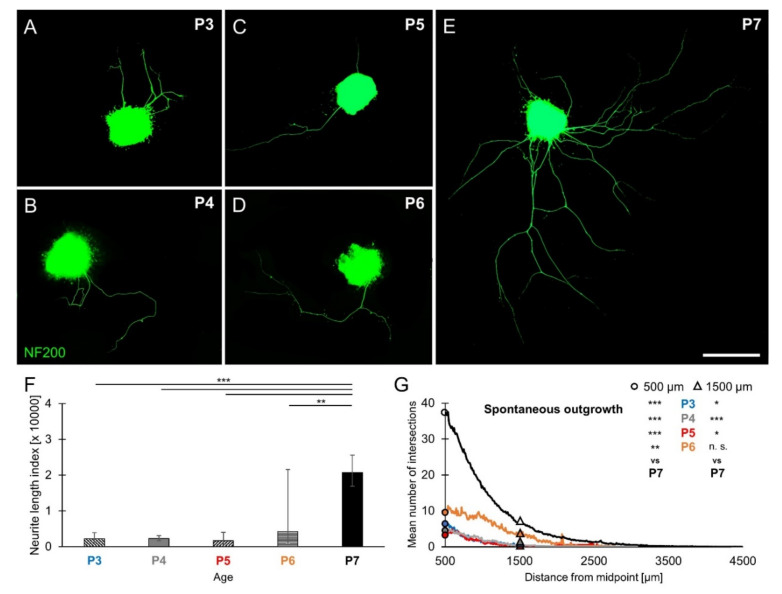
Spontaneous neurite outgrowth in spiral ganglion explants (SGEs) from P3 to P7 mouse cochlea. P3–P7 SGEs were cultured in medium devoid of neurotrophic supplements. After 4 days in vitro (DIV), SGEs were fixed and stained for NF200. Neurite outgrowth was quantified and expressed as neurite length index (NLI) and the number of extending neurites was approximated by the mean number of intersections. (**A**–**E**) Images of NF200-immunolabeled (green) P3–P7 SGEs are shown. Scale bar (A–E): 500 µm. (**F**) Neurite outgrowth was quantified. Bars represent geometric mean NLI values in µm and error bars depict ± the upper and lower 95% confidence intervals for the mean. (**G**) To compare neurite number and extension, histograms of the mean number of intersections were plotted as a function of the distance from the midpoint of the SGE. The mean (arithmetic mean) numbers of intersections at 500 µm (circles) and 1500 µm (triangles) distance from midpoint for the different age groups P3–P6 were statistically compared to P7 results; one-way ANOVA, Dunnett post-hoc test, n. s. = not significant (*p* > 0.05), * *p* < 0.05, ** *p* < 0.01, *** *p* < 0.001.

Age-dependent differences in spontaneous neurite outgrowth were investigated in P3, P4, P5, P6, and P7 SGEs cultured in medium devoid of neurotrophic supplements. SGEs extended a small number of neurites radially (Figure 1A–E). As shown in Figure 1A–D, very few neurites were found in P3–P6 SGEs. P7 SGEs extended a substantially larger number of neurites (Figure 1E). The overall neurite outgrowth performance of individual SGEs was quantified by computing the cumulated neurite length index (NLI) with a modified Sholl analysis routine (see methods section). In addition to the NLI value, the mean number of intersections at 500 µm distance from the midpoint of the explant could be extracted, representing an estimate for the number of extending neurites. The overall neurite outgrowth (Figure 1F) in P7 SGEs resulted in an NLI of 20,774 (±4811/3909; *n* = 103), which was significantly larger than the NLI obtained in P3 (2186 ± 1722/964; *n* = 15), P4 (2257 ± 826/605; *n* = 37), P5 (1696 ± 2332/981; *n* = 16), and P6 SGEs (4238 ± 17,339/3405; *n* = 7). As shown in Figure 1G, the mean number of intersections at 500 µm distance (circles) amounted to 37.43 (±29.56; *n* = 67) in P7 SGEs. The mean number of intersections in P7 SGEs was significantly larger than that observed at P3 (6.86 ± 12.84; *n* = 14), P4 (4.8 ± 4.92; *n* = 25), P5 (3.8 ± 4.89; *n* = 15), and P6 (9.57 ± 12.26; *n* = 7). The mean number of intersections at 1500 µm distance (triangles) representing an estimate for the number of long-range neurites amounted to 6.40 (± 9.39; *n* = 67) in P7 SGEs, which was significantly larger than that observed at P3 (0.36 ± 1.08; *n* = 14), P4 (0.40 ± 0.87; *n* = 25), and P5 (0.27 ± 1.03; *n* = 15). A mean number of 4.00 ± 5.92 (*n* = 7) intersections was obtained in P6 SGEs, which was not significantly different from P7, indicating a pronounced neurite extension in P6 and P7 SGNs. These findings demonstrated an age-dependent increase in spontaneous neurite outgrowth of SGEs from P3 to P7 mouse cochlea, both in neurite number and extension.

### 3.2. Age-Dependent Differences in BDNF-Stimulated Neurite Outgrowth

**Figure 2:**
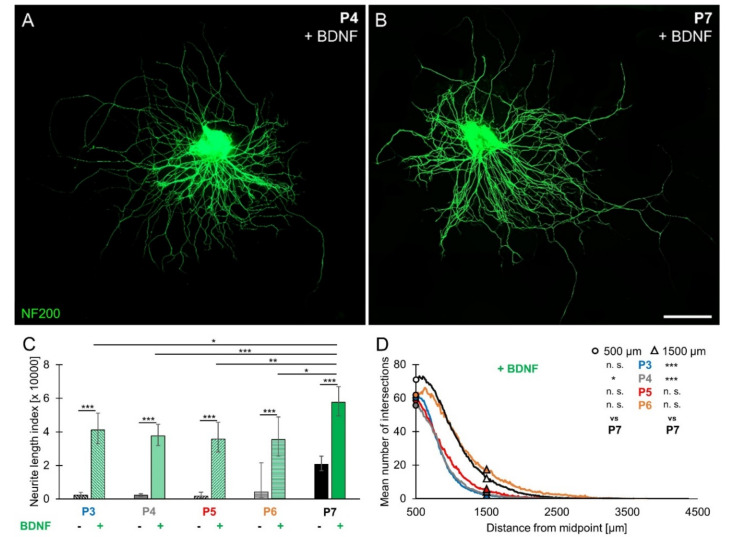
Brain-derived neurotrophic factor (BDNF)-stimulated neurite outgrowth in spiral ganglion explants from P3 to P7 mouse cochlea. P3–P7 SGEs were cultured in medium supplemented with 25 ng/mL BDNF (1.85 nM). After 4 DIV, SGEs were fixed and stained for NF200. Neurite outgrowth was quantified and the number of extending neurites was determined. (**A,B**) Images of NF200-immunolabeled (green) P4 (**A**) and P7 (**B**) SGEs are shown. Scale bar (**A,B**): 500 µm. (**C**) Neurite outgrowth was quantified. BDNF-stimulated neurite outgrowth was statistically compared to spontaneous neurite outgrowth (columns with filling patterns) in P3–P7 SGEs. Bars represent geometric means in µm; error bars indicate ± upper and lower 95% confidence intervals for the mean. (**D**) To compare neurite number and extension, histograms of the number of intersections were plotted as a function of the distance from the midpoint. The mean (arithmetic mean) numbers of intersections at 500 µm (circles) and 1500 µm (triangles) distance from midpoint for the different age groups P3–P6 were statistically compared to P7 results; two-way ANOVA, Dunnett post-hoc test (to compare different ages) or Tukey–Kramer post-hoc test (to compare different treatments), n. s. = not significant (*p* > 0.05), * *p* < 0.05, ** *p* < 0.01, *** *p* < 0.001.

Age-dependent differences in responsiveness to BDNF were investigated in SGEs cultured in medium supplemented with 25 ng/mL BDNF (1.85 nM). As observed for spontaneous neurite outgrowth, SGEs extended neurites radially. Images of BDNF-treated P4 and P7 SGEs are shown in Figure 2A,B. Quantification of neurite outgrowth (Figure 2C) revealed that BDNF-stimulated neurite outgrowth was significantly larger than spontaneous neurite outgrowth at all ages. Supplementation of BDNF to P7 SGEs resulted in an NLI of 57,596 (±9085/7845; *n* = 108), which was significantly larger than the NLI obtained for P3 (41,117 ± 5420/4784; *n* = 50), P4 (37,614 ± 7044/5933; *n* = 87), P5 (35,699 ± 9144/7281; *n* = 40), and P6 SGEs (35,410 ± 28,505/15,789; *n* = 23). As shown in Figure 2D, the mean number of intersections at 500 µm distance (circles) amounted to 70.17 (±40.36; *n* = 69) in P7 SGEs. Significantly fewer intersections were counted in P4 SGEs (55.24 ± 30.31; *n* = 70). However, the mean number of intersections in P3 (61.72 ± 24.47; *n* = 50), P5 (59.13 ± 34.29; *n* = 40), and P6 SGEs (60.96 ± 49.68; *n* = 23) did not significantly differ from the mean number of intersections in P7 SGEs. At 1500 µm distance (triangles), the mean number of intersections amounted to 12.45 (±19.12; *n* = 69) in P7 SGEs, which did not significantly differ from the mean number of intersections in P6 (17.87 ± 40.45; *n* = 23) and P5 SGEs (5.68 ± 8.83; *n* = 40), but was significantly larger compared to P3 (2.52 ± 2.80; *n* = 50) and P4 SGEs (3.20 ± 3.73; *n* = 70). As observed for spontaneous neurite outgrowth, BDNF-stimulated neurite outgrowth increased between P3 and P7 and was most pronounced in SGEs from P7 mouse cochleae.

### 3.3. Age-Dependent Differences in Small-Molecule TrkB Agonist-Stimulated Neurite Outgrowth

**Figure 3:**
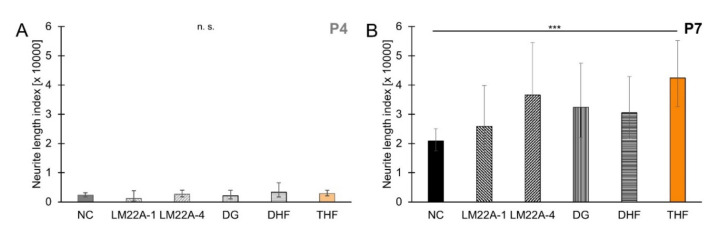
Small-molecule tropomyosin receptor kinase B (TrkB) agonist-stimulated neurite outgrowth in spiral ganglion explants from P4 and P7 mouse cochlea. P4 (**A**) and P7 (**B**) SGEs were cultured in medium devoid of neurotrophic compounds (NC) or in medium supplemented with 1 µM LM22A-1, LM22A-4, deoxygedunin (DG), 7,8-dihydroxyflavone (DHF), or 7,8,3′-trihydroxyflavone (THF). After 4 DIV, SGEs were fixed and stained for NF200. Neurite outgrowth was quantified. Bars represent geometric means in µm; error bars indicate ± upper and lower 95% confidence intervals for the mean; one-way ANOVA, Dunnett post-hoc test, n. s. = not significant (*p* > 0.05), *** *p* < 0.001.

Age-dependent differences in responsiveness to small-molecule TrkB agonists were tested using five compounds that were previously described to exert neurotrophic effects [18,19,20,23,24]. SGEs from P4 and P7 cochleae were cultured in medium supplemented with 1 µM of any of these five small-molecule TrkB agonists. As shown in Figure 3A, neurite outgrowth in small-molecule TrkB agonist-treated P4 SGEs did not significantly differ from spontaneous neurite outgrowth when no compound was applied. Spontaneous neurite outgrowth resulted in an NLI of 2288 (±788/586; *n* = 39). This NLI was not significantly different from the NLI obtained with supplement of LM22A-1 (1167 ± 2599/805; *n* = 9), LM22A-4 (2618 ± 1375/902; *n* = 11), DG (2063 ± 1876/983; *n* = 11), DHF (3324 ± 3263/1647; *n* = 13), or THF (2846 ± 1121/804; *n* = 20). In P7 SGEs, supplementation of small-molecule TrkB agonists resulted in stimulated neurite outgrowth compared to spontaneous neurite outgrowth (20,886 ± 4118/3440; *n* = 104). However, as shown in Figure 3B, neurite outgrowth with supplementation of LM22A-1 (25,859 ± 13,997/9079; *n* = 18), LM22A-4 (36,537 ± 18,001/12,057; *n* = 21), DG (32,375 ± 15,093/10,295; *n* = 23), and DHF (30,479 ± 12,376/8802; *n* = 29) did not significantly differ from spontaneous neurite outgrowth. Only THF (42,402 ± 12,716/9781; *n* = 49) significantly stimulated neurite outgrowth in P7 SGEs, identifying this small-molecule TrkB agonist as a stimulator of neurite outgrowth in SGEs from P7 mouse cochlea, while no responsiveness to THF and other small-molecule TrkB agonists was observed in SGEs from P4 mouse cochlea.

### 3.4. Age-Dependent Differences in THF Efficacy

**Figure 4:**
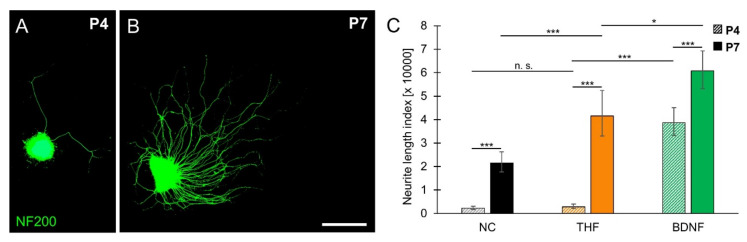
**Age-dependent efficacy of THF.** SGEs from P4 and P7 mouse cochlea were cultured in medium devoid of neurotrophic compounds (NC) or in medium supplemented with 1 µM THF or 25 ng/mL BDNF. After 4 DIV, SGEs were fixed and stained for NF200. (**A**,**B**) Images of THF-treated NF200-immunolabeled (green) P4 (A) and P7 (B) SGEs are shown. Scale bar (A,B): 500 µm. (**C**) Neurite outgrowth was quantified and statistically compared for P4 (patterned bars) and P7 (solid bars) SGEs. Bars represent geometric means in µm; error bars indicate ± upper and lower 95% confidence intervals for the mean; one-way ANOVA, Dunnett post-hoc test (to compare different treatments) or Tukey–Kramer post-hoc test (to compare different ages); n. s. = not significant (*p* > 0.05), * *p* < 0.05, *** *p* < 0.001.

Age-dependent differences in responsiveness to THF were further characterized in SGEs from P4 and P7 cochlea. Neurite outgrowth in SGEs cultured in medium supplemented with 1 µM THF is shown in Figure 4A,B. To evaluate age-dependent differences in THF-stimulated neurite outgrowth, neurite outgrowth was related to spontaneous and BDNF-stimulated neurite outgrowth. As shown in Figure 4C, THF-stimulated neurite outgrowth, which resulted in an NLI of 2846 (±1121/804; *n* = 20), did not significantly differ from spontaneous neurite outgrowth (2288 ± 788/586; *n* = 39) in P4 SGEs. THF-stimulated neurite outgrowth was significantly smaller than BDNF-stimulated neurite outgrowth (38,626 ± 6404/5490; *n* = 103). In P7 SGEs, similar to supplementation of BDNF (60,749 ± 8562/7514; *n* = 129), supplementation of THF resulted in an NLI of 41,575 (±10,773/8553; *n* = 59), which was significantly larger than the NLI obtained for spontaneous neurite outgrowth (21,563 ± 4661/3833; *n* = 114). These results indicated that the small-molecule TrkB agonist THF can only partially imitate the effect of BDNF in SGEs from P7 mouse cochlea, but not in SGEs from P4 mouse cochlea, demonstrating an age-dependent efficacy of THF.

### 3.5. THF Efficacy with Low Concentrations of BDNF and NT-3

**Figure 5:**
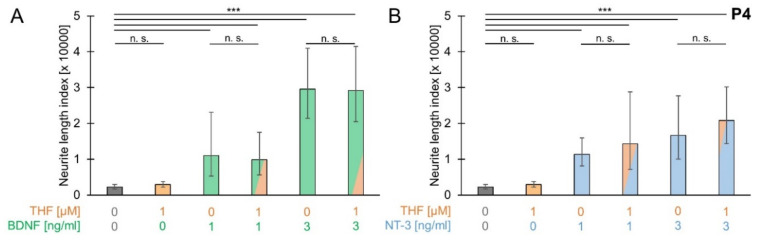
THF efficacy with low concentrations of BDNF and NT-3. SGEs from P4 cochleae were cultured in medium supplemented with 1 µM 7,8,3′-trihydroxyflavone (THF) and/or BDNF (**A**) or NT-3 (**B**) or in medium devoid of neurotrophic compounds. BDNF and NT-3 were tested at low concentrations of 1 and 3 ng/mL and were applied simultaneously with THF or alone. After 4 DIV, SGEs were fixed and stained for NF200. Neurite outgrowth was quantified. Bars represent geometric means in µm; error bars indicate ± upper and lower 95% confidence intervals for the mean; one-way ANOVA, Tukey–Kramer post-hoc test, n. s. = not significant (*p* > 0.05), *** *p* < 0.001.

To investigate whether THF efficacy involves additional factors which are differentially expressed during postnatal development, SGEs from P4 mouse cochlea were cultured in medium supplemented with 1 µM THF and low concentrations (1 ng/mL or 3 ng/mL) of either BDNF or NT-3. As shown in Figure 5A, simultaneous application of THF and BDNF resulted in an NLI of 9912 (±7639/4314; *n* = 10) with 1 ng/mL BDNF and 29,168 (±12,346/8675; *n* = 23) with 3 ng/mL BDNF supplement. Neurite outgrowth for both conditions was not significantly different from neurite outgrowth when only 1 ng/mL BDNF (11,046 ± 12,032/5759; *n* = 10) or 3 ng/mL BDNF (29,596 ± 11,368/8216; *n* = 26) was supplemented. Under all conditions, with supplementation of BDNF or NT-3, neurite outgrowth was significantly larger than spontaneous neurite outgrowth (2253 ± 757/566; *n* = 41), whereas neurite outgrowth with supplementation of THF (2931 ± 878/675; *n* = 27) did not significantly differ from spontaneous neurite outgrowth. Consistent findings were obtained for supplementation of NT-3. As shown in Figure 5B, simultaneous application of THF and NT-3 resulted in an NLI of 14,324 (±14,450/7193; *n* = 8) with 1 ng/mL NT-3 and 20,803 (±9355/6452; *n* = 22) with 3 ng/mL NT-3 supplement. Neurite outgrowth with simultaneous application of THF and NT-3 was not significantly larger than neurite outgrowth when only 1 ng/mL NT-3 (11,379 ± 4554/3252; *n* = 21) or 3 ng/mL NT-3 (16,693 ± 11,053/6649; *n* = 18) was supplemented. These findings indicated that BDNF and NT-3 did not affect THF efficacy.

### 3.6. THF Efficacy with Compounds Released from the Organ of Corti

**Figure 6:**
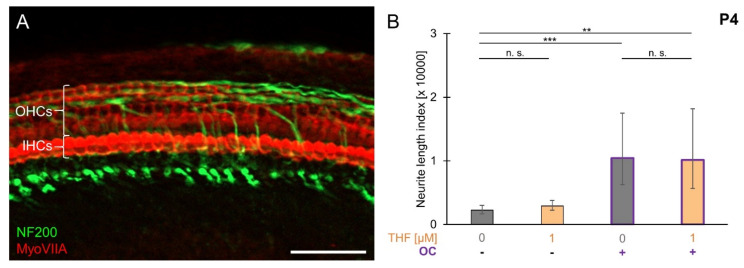
THF efficacy with compounds released from the organ of Corti. SGEs from P4 cochleae were cultured in medium supplemented with 1 µM THF or devoid of neurotrophic compounds. Additionally, SGEs were cultured together with whole P4 OC explants in a spiral ganglion/organ of Corti (SG/OC) co-culture. SG/OC co-cultures were cultured in medium devoid of neurotrophic compounds or supplemented with 1 µM THF. (**A**) A detailed image of a P4 OC explant fixed and stained directly after dissection is shown. OC explants were stained for NF200 (green: neuronal fibers) and MyoVIIA (red: hair cells). Scale bar: 50 µm. (**B**) After 4 DIV, explants were fixed and stained for NF200. Neurite outgrowth from SGEs was quantified. Bars represent geometric means in µm; error bars indicate ± upper and lower 95% confidence intervals for the mean one-way ANOVA, Tukey–Kramer post-hoc test, n. s. = not significant (*p* > 0.05), ** *p* < 0.01, *** *p* < 0.001.

To investigate whether organ of Corti (OC)-derived compounds affect THF efficacy, SGEs were co-cultured with OC explants from P4 mouse cochlea. The full integrity of the OC was confirmed by MyoVIIA and NF200 staining. As shown in Figure 6A, demolished peripheral processes remained in-between three rows of outer hair cells (OHCs) and one row of inner hair cells (IHCs). In the spiral ganglion/organ of Corti (SG/OC) co-culture, quantification of neurite outgrowth (Figure 6B) resulted in an NLI of 10,468 (±7043/4210; *n* = 22), which was significantly larger than the NLI obtained when SGEs were cultured separately (2253 ± 757/566; *n* = 41), indicating a release of neurotrophic compounds from OC explants. Supplementation of 1 µM THF to the SG/OC co-culture resulted in an NLI of 10,153 (±8019/4480; *n* = 6), a value not different from the SG/OC co-culture. These findings indicated that neurotrophic compounds released from P4 organ of Corti did not affect THF efficacy.

### 3.7. Trk Receptor and Ligand Involvement in Neurite Outgrowth

**Figure 7:**
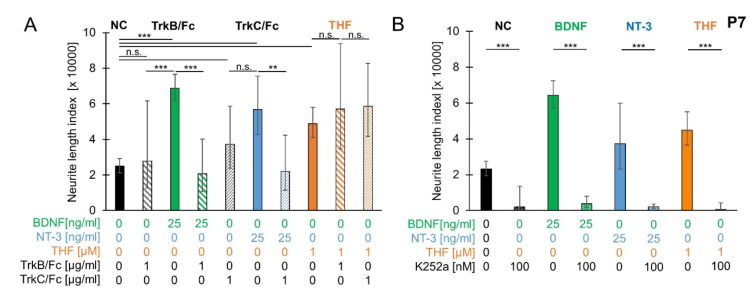
Trk receptor and ligand involvement in neurite outgrowth. (**A**) SGEs from P7 mouse cochlea were cultured in medium supplemented with 1 µg/mL TrkB ligand scavengers (recombinant TrkB/Fc or TrkC/Fc), 25 ng/mL neurotrophin (BDNF or NT-3), or both scavenger and neurotrophin in combination. TrkB/Fc: TrkB/Fc was applied alone and simultaneously with BDNF. TrkC/Fc: TrkC/Fc was applied alone and simultaneously with NT-3. THF: 1 µM THF was applied alone or in combination with either TrkB/Fc or TrkC/Fc. After 4 DIV, SGEs were fixed and stained for NF200. Neurite outgrowth was quantified. (**B**) P7 SGEs were cultured in medium supplemented with 100 nM K252a alone or with K252a and 25 ng/mL BDNF, 25 ng/mL NT-3, or 1 µM THF. For comparison, compounds were applied alone and SGEs were cultured in medium devoid of neurotrophic supplements. After 4 DIV, SGEs were fixed and stained for NF200. Neurite outgrowth was quantified. Bars represent geometric means in µm; error bars indicate ± upper and lower 95% confidence intervals for the mean; one-way ANOVA, Tukey–Kramer post-hoc test, n. s. = not significant (*p* > 0.05), * *p* < 0.05, ** *p* < 0.01, *** *p* < 0.001.

To test whether the age-dependent increase in BDNF-stimulated neurite outgrowth in SGEs from P7 mouse cochlea was attributable to BDNF alone, we tested whether recombinant TrkB/Fc proteins, which act as TrkB ligand scavengers, can inhibit BDNF function. P7 SGEs were cultured in medium supplemented with 25 ng/mL BDNF and 1 µg/mL TrkB/Fc. As shown in Figure 7A, simultaneous application of TrkB/Fc and BDNF resulted in an NLI of 20,702 (±19,430/10,024; *n* = 13). Neurite outgrowth was significantly reduced compared to neurite outgrowth with BDNF supplementation (76,623 ± 7833/7032; *n* = 145). Neurite outgrowth with supplementation of TrkB/Fc alone (27,838 ± 33,708/15,246; *n* = 13) was not significantly different from spontaneous neurite outgrowth (24,878 ± 4301/3668; *n* = 133), indicating that spontaneous neurite outgrowth in P7 SGEs may not be mediated by TrkB ligands. Likewise, the age-dependent increase in BDNF-stimulated neurite outgrowth was rather not attributed to an increase in responsiveness to BDNF.

To test whether TrkC receptor ligands cause spontaneous neurite outgrowth in P7 SGEs, recombinant TrkC/Fc proteins, which act as TrkC ligand scavengers, were applied. 1 µg/mL TrkC/Fc was supplemented simultaneously with 25 ng/mL NT-3. NT-3 supplementation alone resulted in an NLI of 56,795 (±18,742/14,092; *n* = 14), which was significantly larger than the NLI obtained for spontaneous neurite outgrowth (24,878 ± 4301/3668; *n* = 133). Simultaneous application of TrkC/Fc and NT-3 resulted in an NLI of 22,048 (±20,297/10,569; *n* = 14), which was significantly smaller than the NLI obtained for supplementation of NT-3. Neurite outgrowth with TrkC/Fc supplement only (37,321 ± 21,303/13,562; *n* = 7) was not significantly (*p* = 0.1982) different from spontaneous neurite outgrowth (24,878 ± 4301/3668; *n* = 133). These findings indicated that spontaneous neurite outgrowth was not caused by TrkC ligands.

To probe whether the pronounced THF-stimulated neurite outgrowth was mediated by Trk receptors, P7 SGEs were supplemented with 1 µM THF and 1 µg/mL of either TrkB/Fc or TrkC/Fc. THF applied alone significantly increased neurite outgrowth to an NLI of 48,839 (±9215/7752; *n* = 72). In contrast to the BDNF and NT-3 inhibition seen after simultaneous application of the Trk ligand scavengers TrkB/Fc or TrkC/Fc, respectively, their combination with THF failed to reduce the outgrowth back to control level. The addition of TrkB/Fc or TrkC/Fc to THF resulted in NLI values of 57,065 (±36,848/22,390; *n* = 7) and 58,688 (±24,007/17,037; *n* = 17), respectively, which were not statistically different from THF-induced outgrowth. Additionally, we investigated the effect of the pan-Trk inhibitor K252a [37] by supplementing 100 nM alone or simultaneously with 25 ng/mL BDNF, 25 ng/mL NT-3, or 1 µM THF. As shown in Figure 7B, supplementation of K252a resulted in an NLI of 1920 (±11,654/1648; *n* = 3), which was significantly lower than the NLI obtained for spontaneous neurite outgrowth (23,193 ± 4444/3726; *n* = 136). Simultaneous application of K252a and 25 ng/mL BDNF or 25 ng/mL NT-3 resulted in an NLI of 3832 (±4187/2001; *n* = 7) and 2090 (±1574/898; *n* = 3), respectively. Neurite outgrowth for supplementation with K252a and a neurotrophin was significantly weaker than neurite outgrowth when BDNF (64,398 ± 8146/7237; *n* = 150) or NT-3 (37,196 ± 22,604/14,060; *n* = 18) was applied alone. Likewise, simultaneous application of K252a and 1 µM THF resulted in an NLI of 607 (±3636/520; *n* = 3), which was significantly lower than the NLI obtained for supplementation of THF (44,898 ± 10,233/8330; *n* = 74), demonstrating that K252a significantly and nearly completely inhibited neurite outgrowth under all conditions. These results indicated that Trk receptors were crucial in mediating neurite outgrowth and THF efficacy in P7 SGEs.

## 4. Discussion

In the present study, we observed an age-dependent increase in spontaneous and BDNF-stimulated neurite outgrowth in postnatal SGEs from P3 to P7 mouse cochlea. During the same period, age-dependent differences in the efficacy of the small-molecule TrkB receptor agonist THF were observed. While BDNF and NT-3 significantly stimulated neurite outgrowth in P4 SGEs, the small-molecule TrkB agonist THF (and four other small-molecule TrkB agonists) at a concentration of 1 µM could not mimic the effect of BDNF P4 SGEs. However, THF could significantly stimulate neurite outgrowth in P7 SGEs, but to a lesser extent than BDNF or NT-3.

### 4.1. Age-Dependent Differences in Neurite Outgrowth

Our findings are in accordance with observations made in embryonic day 18 (E18) to P20 rat SGEs, as neurite number and length were found to be increased in P5 and P10 rat SGEs [25]. Kondo et al. claimed that, while the increase in spontaneous neurite outgrowth may result from a decreasing dependence on neurotrophic support with age, the increase in BDNF-stimulated neurite outgrowth may result from an increased responsiveness to BDNF. In the present study, we found that BDNF-stimulated neurite outgrowth, which amounted to an NLI of 57,596 (*n* = 108) in P7 SGEs, was about 20,000 units bigger than the NLI of BDNF-stimulated P3–P6 SGEs. These 20,000 units resembled exactly the extent of spontaneous neurite outgrowth that was seen in P7 SGEs and which amounted to 20,774 (*n* = 103). The addition of 25 ng/mL BDNF increased the neurite outgrowth performance of the SGEs of about 35,000 units, independently of the age of the explants used (from P3 to P7), ranging from 31,172 units for P6 to 38,931 units for P3. These findings may allow the prediction that the age-dependent increase in BDNF-stimulated neurite outgrowth is not mediated by an increased responsiveness of SGNs to BDNF with age. Furthermore, we showed that an excess of the TrkB and TrkC ligand scavengers TrkB/Fc and TrkC/Fc completely diminished the increased responsiveness of SGNs to BDNF and NT-3, but did not reduce spontaneous neurite outgrowth. Similar results have been shown for Trk/Fc scavenger experiments in embryonic primary hippocampal neurons [44] and adult retinal ganglion cell cultures [45]. Therefore, we assumed that the increased spontaneous neurite outgrowth in P7 SGEs compared to earlier postnatal stages was not caused by BDNF or NT-3, but might involve additional, presently unidentified factors. 

It is generally believed that SGN survival requires sustained neurotrophic support (for review, see [46]), whereby sources for neurotrophic support change with developmental changes. While neurotrophin release from the OC is downregulated during the first postnatal week [33,36,47], neurotrophin expression is upregulated in the auditory brainstem [48,49] and in SGNs themselves [33], thereby providing alternative sources for neurotrophic support. Since we did not observe an increase in neurite outgrowth before P7 and the afferent innervation pattern reaches mature organization as early as P6 in mice [50], it appears unlikely that the observed effect is associated with target-finding of afferent SG dendrites, but likely coincides with other developmental changes. Upon target innervation, vestibular ganglion neurons were found to switch their responsiveness from BDNF to glial cell line-derived neurotrophic factor (GDNF) [51], whereby changes in sensitivity are supposed to be regulated by changes in available factors. We assumed that similar processes may occur in SGNs when BDNF or NT-3 expression levels decline, as several other growth factors are accessible to SGNs (for review, see: [52,53]). Members of the transforming growth factor beta (TGF-β) superfamily [54,55,56], GDNF [57,58], artemin [59], and ciliary neurotrophic factor (CNTF) [60,61,62] were found to significantly stimulate the survival of SGNs. GDNF [63,64], CNTF [60,62], and acidic (aFGF = FGF-1) and basic (bFGF = FGF-2) fibroblast growth factors [65,66] were seen to significantly stimulate neurite outgrowth. For TGF-β1, GDNF, and artemin, differential expression patterns in response to injury and deafening injury were described [64,67,68], whereas GDNF and FGF-1 were found to be differentially expressed during development [64,68]. Coinciding with the increased neurite outgrowth at the end of the first postnatal week, FGF-1 expression was found to be upregulated between P6 and P14 in rat SGNs [69], making FGF-1 a promising candidate which will be tested in future experiments. Selective inhibition of receptors and/or signaling pathways may help to shed light on the involvement of distinct growth factors in stimulated neurite outgrowth observed in SGEs from P7 mouse cochlea.

### 4.2. Neurotrophic Effects Elicited by Small-Molecule TrkB Agonists

Although BDNF significantly stimulated neurite outgrowth in SGEs from P4 cochlea, we found that none of the five small-molecule TrkB agonists, including LM22A–1, LM22A–4, DG, DHF, and THF, were able to stimulate neurite outgrowth beyond control levels. In mouse SGEs, THF efficacy was dependent on the developmental stage. Given that THF did not stimulate neurite outgrowth in P4 SGEs, we claim that THF alone is not sufficient to elicit neurotrophic effects in SGNs. However, both DHF and THF were found to stimulate survival and neurite outgrowth in SGNs when tested in P1 and P2 organotypic cochlear cultures or in vivo [23,24]. Based on our results and the findings of Yu et al., we tested whether small-molecule efficacy may be facilitated by additional factors which are differentially expressed in the surrounding tissue and/or in SGNs themselves. By supplementing BDNF or NT-3 and co-culturing OC explants, we did not find evidence that THF efficacy in SGNs depends on factors released by the OC (when cultured in vitro). As we observed differences in THF efficacy in SGE cultures, the critical factor is likely (also) upregulated within the spiral ganglion itself. Given that the age-dependent efficacy of THF coincided with the age-dependent increase in spontaneous and BDNF-stimulated neurite outgrowth, it seems likely that the same factor(s) which stimulate(s) neurite outgrowth also facilitates THF efficacy. Further experiments are needed to unravel the molecular mechanisms underlying THF efficacy. Experiments in which THF is simultaneously applied with one of the growth factors discussed above may help to identify the critical factor causing THF efficacy.

### 4.3. Molecular Mechanism Underlying Age-Dependent Differences in Neurite Outgrowth and THF Efficacy

Up to this point, we assumed that the observed age-dependent differences in neurite outgrowth and THF efficacy may be caused by the same unknown, presently unidentified factors and/or molecular mechanisms. As THF failed to mimic BDNF function in SGEs from P4 mouse cochlea, we claim that THF does not act like BDNF and is not a full TrkB agonist. Contradictory findings on TrkB receptor activation by small-molecule TrkB agonists have been reported before. Studies on the phosphorylation of TrkB and its downstream signaling molecules stimulated by LM22A–1 and LM22A-4 [18], DG [19], DHF, and THF [20,21,24] were performed, coming to the point that all of these small-molecule TrkB agonists act via TrkB. By challenging the efficacy of LM22A-4 and DHF in various recombinant reporter assays, with CellSensor^®^ NFAT-bla CHO-K1 cell lines, and a primary cortico-striatal co-culture, Todd et al. did not observe phosphorylation of TrkB, AKT (also known as protein kinase B (PKB)), or ERK (extracellular signal-regulated kinase), claiming a multi-faceted action of small-molecule compounds, which likely involves pathways independent of TrkB [70]. For 25 ng/mL BDNF and 25 ng/mL NT-3, the use of 1 µg/mL TrkB/Fc and TrkC/Fc, respectively, was sufficient to block the neurotrophin effect on neurite outgrowth. While K252a was able to fully block the THF effect on neurite outgrowth at P7, the scavengers failed to do so. On the one hand, based on excessively large differences in molecule size and molarity between the applied 1 µM THF and 1 µg/mL TrkB/Fc or TrkC/Fc, representing approximately 7.7 nM and 7.1 nM, respectively, we cannot rule out the effectiveness of THF in these experiments. It is very likely that the number of TrkB/Fc molecules was too low to scavenge enough THF molecules to test the involvement of TrkB receptors on THF action. Having said that, we cannot rule out the possibility that THF and BDNF utilize different mechanisms to activate TrkB either, as it was shown for DHF [71]. Thus, selective inhibition of TrkB receptors is necessary to test TrkB receptor involvement. Selective TrkB inhibitors like cyclotraxin-B [72] or ANA-12 [73], inhibitory antibodies targeting TrkB [18,74], and/or conditional TrkB knockout mice [75] may be used to challenge TrkB involvement. Using conditional TrkB knockout mice, it has been shown that functional blockage of TrkB abolishes the neurotrophic effect of DHF [19] and THF [23,24] in vivo, whereby the involvement of the endogenous ligand BDNF was ruled out by experiments performed on conditional BDNF knockout mice [19]. However, as neurotrophic effects observed in P7 SGEs were completely blocked by supplementing the pan-Trk inhibitor K252a, we assumed that Trk receptors were involved in pronounced neurite outgrowth in P7 SGEs. Moreover, an excessive blockage of neurite outgrowth below the spontaneous outgrowth level was seen in P7 explants; we assigned this phenomenon to the non-selective nature of the protein kinase inhibitor K252a. It is also possible, that THF action can be attributed to other BDNF targets such as the pan-neurotrophin receptor p75^NTR^ [76,77]. The main functions associated with p75^NTR^ are the regulation of cell survival, axonal growth, cell cycle, and myelin formation [78,79]. The delicate interplay between the different neurotrophin receptors and their multiple downstream signaling pathways is a complex affair [80,81,82], which might play a crucial role in this context. Given that the spontaneous neurite outgrowth was not affected by scavenging TrkB and TrkC ligands, we claim a BDNF/NT-3 independent neurite outgrowth in SGEs from P7 mouse cochlea. Based on the general assumption that TrkA receptors are not present in SGNs [6,83,84] (for review, see [85]), one has to assume that TrkB activation may involve molecular mechanisms induced by other receptors and/or signaling pathways. Highlighting the possible interaction of different receptors and signaling pathways, collaboration of GDNF-mediated with TrkA-mediated signaling pathways was observed before [86]. Various combinations of growth factors were found to synergistically act on SGNs. Members of the TGF-β superfamily were found to potentiate neurotrophic effects mediated by neurotrophins [54,55,56]. Likewise, co-administration of FGF-2 and GDNF provided synergistic support on SGN survival [87]. When supplementing a combination of BDNF ([60,62] or NT-3 [88,89] and CNTF in vitro, neuronal survival and neurite outgrowth were significantly higher in SGNs treated with a combination of the two growth factors compared to treatment with each factor alone. Highlighting the possibility that growth factors may alter the expression of other growth factors and/or receptors, TGF-β1 was found to modulate the expression of FGF-2 mRNA [90]. Identifying the currently unidentified factors(s) involved in the BDNF/NT-3-independent neurite outgrowth in SGEs from P7 mouse cochlea and selective inhibition of distinct signaling molecules will help to investigate the molecular mechanisms underlying THF-mediated activation of TrkB in SGNs.

## 5. Conclusions

Our studies revealed that, in contrast to BDNF and NT-3, THF failed to stimulate neurite outgrowth in P4 SGEs. In P7 SGEs supplementation of THF significantly stimulated neurite outgrowth, demonstrating an age-dependent efficacy of THF. Likewise, an age-dependent increase in spontaneous and BDNF-stimulated neurite outgrowth across postnatal development was observed. Scavenging of TrkB and TrkC ligands did not diminish the age-dependent increase in neurite outgrowth, indicating that age-dependent differences were not caused by BDNF and NT-3. Based on our findings, we assume that neurite outgrowth and THF efficacy in postnatal SGNs involve other, currently unidentified factors and/or molecular mechanisms. These mechanisms, underlying small-molecule efficacy need to be carefully investigated in various experimental settings to disclose differences compared to molecular mechanisms induced by the original compound. To eliminate the spatial gap between SGNs and CIs, the understanding of developmental processes and small-molecule efficacy is an indispensable prerequisite to develop novel therapeutic strategies for stimulating neurite outgrowth in SGNs and improving CI performance.
